# A Decision-Making Algorithm for Rearchitecting of Healthcare Facilities to Minimize Nosocomial Infections Risks

**DOI:** 10.3390/ijerph17030855

**Published:** 2020-01-30

**Authors:** Yasaman Parsia, Shahryar Sorooshian

**Affiliations:** 1 Independent Researcher, 405 30 Gothenburg, Sweden; parsiayasi@gmail.com; 2 Department of Business Administration, University of Gothenburg, 405 30 Gothenburg, Sweden

**Keywords:** nosocomial infection, healthcare facility, risky department(s), rearchitecting, multiple criteria decision making

## Abstract

Most of the healthcare facilities (HFs) have to face the nosocomial infections (NIs), which increase the rates of morbidity, mortality, and financial burden on the HFs and the patients. The control of the NIs is a global issue and requires additional effort. Because the pathogenic microbes can be transmitted among all the HF departments, the layout and design of the HFs (or the department configuration) is considered to play a significant role in control of the NIs. A few of the departments transmit the microbes more than other departments, called ‘cause’, while some other departments are more infected than others, called ‘effect’. Here, the researchers have stated that both the cause and effect departments are risky. This research tried to propose a comprehensive mathematical algorithm for choosing the high-risk department(s) by applying the NI and the managerial criteria to minimize NIs through rearchitecting of the HFs. To develop the algorithm, the researchers applied the multiple criteria decision-making (MCDM) methods. They used Decision-Making Trial and Evaluation Laboratory (DEMATEL) and modified weighted sum method (WSM) methods, and their hybrid, along with a modified nominal group technique (NGT) for data collection. The proposed algorithm was later validated by implementation in a HF as a case study. Based on all results of the algorithm, the high-risk departments in the HF were identified and proposed to be eliminated from the HF in such a way that the facility would retain its functionality. The algorithm was seen to be valid, and the feasibility of the algorithm was approved by the top managers of the HF after the algorithm was implemented in the case study. In conclusion, the proposed algorithm was seen to be an effective solution for minimizing the NIs risk in every HF by eliminating the high-risk departments, which could simplify the HF manager’s decisions.

## 1. Introduction

A few researchers stated that the healthcare facility (HF) issues, like the hazards and risks noted in HF-related issues, could be due to their bad design [1]. The physical environment of the HFs must consist of an acceptable layout design [2]. Furthermore, the physical design of the HF plays a vital role in controlling the HF-related infections, which increases the incorporation of various measures that can control the infection and decrease the risk of the infection transmission [3]. Moreover, some studies also highlighted the relationship between the spread and transmission of the pathogens or nosocomial infections (NIs) and the architectural design of the HFs [3,4,5,6,7,8]. The NIs, also sometimes called HF-cross infections, are described as a type of infection where the patient is infected during his hospital stay or immediately after he is discharged from the HF [9,10,11,12,13].

One of the main goals related to the design of HFs is the prevention and decrease of NIs [3,14]. The control of Nis improves the functioning of the society [15]. NIs are a major cause of mortality and increase the emotional stress levels and the morbidity rates among the hospitalized patients [16] and even in newborns [17]. Studies have stated that a lot of people are affected by NIs around the world [18,19], and an alarming number of them were infected and eventually died due to NIs [20]. 

A survey of the patients admitted in the acute care hospitals across 30 European countries was carried out during 2011–2012, and it was observed that at any time, a noticeable number of patients had at least one NI [21]. According to a recent estimate, the mean prevalence of the NIs in the European HFs is around 3.2 million patients every year [22]. Therefore, the minimalization of NI risks in HFs should be among the objectives of any society.

Moreover, with respect to an economic viewpoint, the acute care HFs significantly impact the society’s and government’s role as the purchaser and the employer, respectively [14]. NIs lead to an extended hospitalization, which further burden the HFs [13]. For instance, in many of the developed countries, patients suffering from blood-related NIs have to extend their hospital stay by 10–15 days for additional therapy [15,23]. On the other hand, with regard to the HF administrator’s point of view, every patient has to pay some additional fixed (for lighting, air conditioning, etc.) and variable costs. NIs require additional nursing services which would have to be paid by the patients, which further creates an economic burden on the patient [15,24]. In the current business environment, where the performance of the HFs is responsible for their economic stability and compensation, the administrators of such HFs must focus on making decisions to decrease the economic burden caused by the avoidable adverse events, like NIs, which increase the expenses incurred by the HFs and their patients/families [1].

Altering the design of the HFs and the departments housed in these HFs is a strategy which is used for controlling the spread of infections [25]. The construction of the HF buildings is considered to be a dangerous business, since any minor design flaw could become a fresh source of infection [26]. Many evidence-based studies have shown that the design of the HF plays a significant role in reducing the morbidity and incidence rates of the NIs, since many infection control measures are included during the design phase of the HF project [26]. According to Lateef [27], owing to the increased incidence of new and emerging NIs, along with the higher public awareness and expectations, the designing phase of the HF buildings should be paid a lot of attention. Even after the completion of the HF building project, the administrators must put into place a systematic plan for constantly modifying the HF infrastructure [27,28]. 

However, as shown in above paragraphs, the NI risk in HFs exist, and it is not a practical solution to close down the existing HFs because of the extremely high costs of rebuilding [29]. But, after initial completion of the HFs, a systematic and ongoing approach can be adopted to modify, or rearchitect, the infrastructure [27,28]. An extensive search of the different literature databases (such as Web of Science and Scopus), together with interviews with professionals in the field, revealed that there are very few systematic approaches that can be implemented to modify the existing HFs for NI reduction. Hence, the main objective of this study was to formulate an algorithm to rearchitecture HFs to minimize the NI risks by identifying high-risk departments.

A 2020 published systematic literature-review by Parsia and Sorooshian [30] was on the trend of studies on NI, with an analysis of 61,559 articles published since 1915. Based on their work, some research works tried to solve the problem of NIs, but none of them paid attention to department configuration or identified the high-risk departments to remove from HF. In their findings, none of their found research trends/clusters were related to the restructuring or rearchitecting of HFs. In addition, some other references [31,32] called for future research to fill the significant gap of knowledge in the rearchitecting of HFs. 

### Healthcare Facilities Rearchitecting

Whereas rearchitecting is one of a range of strategies that may be implemented to achieve a new structure, it is also a term closely linked with ‘restructuring’ in the minds of many in the workforce [33]. Rearchitecting is a common response to environmental influences, with organizations implementing these changes in order to improve their effectiveness [34]. A rearchitecting plan should be included in the strategic management plan of all organizations, regardless of whether they plan to rearchitect or not [35].

Rearchitecting in HFs started to become more noticeable in the late 1990s, when the United States was still experiencing a decade of almost unprecedented economic prosperity [36]. Referring to the definition by Parsia [37], HF rearchitecting is defined as the decisions targeting the removal of one or several departments in the HF. All HF staff and managers are aware that old structures not only merely fail to serve the patients adequately, but also fail in what even the most reluctant healthcare providers have come to recognize as a medical marketplace [38]. The most obvious product in the medical marketplace is excellence in healthcare, and a facility’s reputation for excellence is a strong incentive to healthcare consumers to select that institution over another [38]. The most important factors that influence the potential consumers are the design of the facility and the patient amenities that the design offers [38]. The first advantage of eliminating extra (high-risk) departments through rearchitecting is to maximize the space in order to enhance flexibility and the capability of handling more patients, and this can be achieved by removing redundant departments (rearchitecting). It also eliminates the unnecessary flows in the clinic area, and the second step is to reorganize all the departments [38]. A number of studies have been published that examine the degree to which rearchitecting, workforce reductions, re-engineering, and the resizing effect the delivery of health services and employee morale [36].

Healthcare facilities have to rearchitect their structures due to technical or financial limitations. These decisions can also be made due to managerial strategies, medical errors, and the rising risk of spreading infections. For many businesses, including those in healthcare, rearchitecting means the loss of employees, positions, departments, or service line [35]. However, its goal is to cut waste, improve profitability, increase productivity, and enhance local, national, or international competitiveness [35,39]. For this research, we used this method minimize infection risks in HFs through rearchitecting. The rearchitecting process, defined as selecting and proposing high-risk departments in HFs to eliminate or increase hygienic policies to minimize NIs risk, helped in this study to make decisions.

## 2. Materials and Methods

### 2.1. Decision-Making and Method Selection for HF Rearchitecting

Decision-making (DM) is a part of every person’s everyday life, with numerous personal and business decisions made daily [40]. However, DM involves the use of knowledge, innovativeness, and insight in order to meet basic needs or address certain issues [41]. A DM process is typically an easy and intuitive task when decision problems having a single criterion are considered [40]. However, when various alternatives or actions with multiple criteria are ranked and assessed, it becomes very complex, and sophisticated methods are then needed [40].

The rearchitecting of a HF requires a decision regarding the selection of high-risk department(s) that will be eliminated from the present processes and operations of the HF. When there are numerous alternatives to choose from, the process of DM requires the evaluation of the decision criteria to ensure that the right choice is selected [41]. Nis risk and managerial criteria were two clusters of criteria which were identified in this study. Selecting high-risk departments from a HF, as a complex organization with multiple criteria, can be run through multiple criteria decision-making (MCDM) methods. Therefore, to choose the correct methods among the many MCDM methods, Gade and Osuri [42] and Parsia [37] described the key aspects to be considered when selecting methods of MCDM, comprised as follows: Ease-of-use, friendly user interface, generalized application domain, robust application, consistent operation, time needed, accuracy of DM course results, technical implementation, application of decision models in sensitivity analyses, and the necessity of fewer human interventions in DM processes.

Accordingly, this study selected two various mathematical levels of MCDM methods, which are enumerated as follows. 

#### 2.1.1. DEMATEL

The Decision-Making Trial and Evaluation Laboratory (DEMATEL) technique was applied in 1974 by Duval, Fontela, and Gabus at the Battelle Memorial Institute, Geneva Research Centre, in order to visualize the structure of complex causal relationships via matrices or digraphs [43,44,45,46]. Among the more powerful DM techniques, the methodology is well-suited for the extraction of interdependent relationships and the intensities of interdependencies, cause and effect, among the complex parts of a system [44,47]. 

DEMATEL utilizes expert knowledge to arrive at a superior understanding of the correlations between factors, in accordance with the relationships and influences among various factors [44]. DEMATEL stems from the graph theory, and the approach involves the conversion of interdependency relationships into cause-and-effect groups using matrices [40,43,45,46,47]. The method can also recognize indirect, direct, and interdependent effects between every complex factor, as well as rank each according to long-term DM strategies, all while indicating scope for improvement [43,45,48,49]. The formulating steps of the DEMATEL can be summed up in five steps, which are based on the efforts of a few researchers [45,50]:

(1) Gathering expert’s opinion and calculating the average matrix Z

As a sample, let us consider a set of m experts and n parameters for this research. Experts are to be allotted a list of factors organized in sets of i and j. They are then to be requested to indicate their assumed degree of impact the factors have on one another (pairwise comparison), that is, how factor i affects factor j. The suggestion can be made in the range of 0 to 4, where 0 implies no influence, 1 implies low influence, 2 implies moderate influence, 3 implies high influence, and 4 implies very high influence. Nonetheless, this scale is only used as an example for this research, or else the rating scale can be according to the researcher’s preference. The amount to which the expert’s perception of factor i affecting factor j is indicated by $X_{ij}$. For each expert, an n×n non-negative matrix is created as $X^{k}=[X_{ij}^{k}]$, in which k is the number of experts participating in the evaluation procedure with 1≤k≤m.

(1) $$X=\begin{array}{cccc} 0 & x_{12} & \cdots & x_{1n}\\ x_{21} & 0 & \cdots & x_{2n}\\ \vdots & \vdots & \ddots & \vdots \\ x_{n1} & x_{n2} & \cdots & 0 \end{array}$$

Every element of the matrix is designated as $X_{ij}$ representing the amount of impact parameter i has on parameter j. An average insight on the experts’ answer has to be attained by calculating the matrix average, which could be termed as an initial direct-relation matrix, shown in Equation (2).

(2) $$Z_{ij}=\frac{1}{m}\overset{m}{\underset{i=1}{\sum }}X_{ij}^{k}$$

(2) Normalizing the initial direct-relation matrix D

In this step, normalized direct-relation matrix D has to be derived from the average matrix Z. This is accomplished by dividing every element by the biggest row sum of Z. The total direct effect on the influence magnitude of the factor with largest direct influence on the other parameters can be given as follows:

(3) $$\underset{0\leq x\leq 1}{\max}\overset{n}{\underset{j=1}{\sum }}Z_{ji}$$

The value of each element in this normalized direct-relation matrix D would vary between zero and one. The computation to obtain the matrix is as shown in Equations (4) and (5):

(4) $$D=\frac{Z}{S}$$

(5) $$S=\underset{0\leq x\leq 1}{\max}\overset{n}{\underset{j=1}{\sum }}Z_{ji}$$

(3) Finding the total relation matrix (T)

This step would realize the total or direct/indirect relationship between each pair of the system factors. The suppositions are that the matrix of indirect influence converges to the null matrix as displayed in Equation (6):

(6) $$\underset{k\to \infty }{\lim}D^{k}=0$$

In the case where 0 is the null matrix with I as an n×n identity matrix, the equation given below holds true:

(7) $$\underset{k\to \infty }{\lim}\left(I+D+D^{2}+\ldots +D^{k}\right)={\left(I-D\right)}^{-1}$$

The matrix of total relation T is, therefore, defined as Equation (8):

(8) $${T=D(I-D)}^{-1}$$

(4) Calculating sums of rows and columns of matrix T

Vector R and C denote the sum of rows and columns respectively in the matrix T. Let vector R be given as n×1 and C be given as 1×n. Therefore, the sum of rows Equation (9) and the sum of columns Equation (10) would be determined as:

(9) $$R=[R_{i}{]}_{n\times 1}=(\overset{n}{\underset{j=1}{\sum }}t_{ij}){}_{n\times 1}$$

(10) $$C=[C_{j}{]}_{n\times 1}=(\overset{n}{\underset{j=1}{\sum }}t_{ij}){}_{n\times 1}$$

(5) Calculate the R−C for all alternatives of matrix T

When i=j, the subtract $\left(R_{i}-C_{j}\right)$ for each factor indicates the net indirect and direct interrelationship that factor i contributes to the system and is given in Equation (11):

(11) $$(R_{i}-C_{j})=\overset{n}{\underset{j=1}{\sum }}t_{ji}-\overset{n}{\underset{k=1}{\sum }}t_{ik}$$

If $\left(R_{i}-C_{j}\right)$ is positive, the influence factor i is a net cause, while if $\left(R_{i}-C_{j}\right)$ is negative, factor i is a net receiver, effect. Based on some researches, practically, the value of (R−C) is more effective and applicable than (R+C) for alternatives prioritization. The component with the highest positive value of (R−C) can be named as the master dispatcher, in the cause group, and the component with the lowest value of (R−C), in the effect group, can be named as the master receiver [51,52,53,54,55,56].

#### 2.1.2. Modified Weighted Sum Method (WSM)

The weighted sum method, known as the traditional multifactor rating scheme, is a very popular, practical, and easily applicable subjective DM process [57,58,59,60,61,62,63,64,65,66,67]. Kumar and Suresh [58] stated that this procedure includes many DM techniques where every alternative could be scored using relevant factors, wherein every factor is weighted based on its significance. In this study, because of the use of different sources of information, management criteria, and infection risk, a modified WSM was used, as presented by Sorooshian and Parsia [59], which was called the WSM, in general, in the study. The authors mentioned x Alternative (A) and y decision Criterion (C) sets, wherein the technique could be algorithmically delineated into five phases as follows:

(1) Initially, decide the priorities for every criterion based on their significance in the DM. Thereafter, after focusing on every priority factor, the weightings (W_x_, %) could be assigned for each criterion, such that the total weight was 100%.
(2) For each alternative, allocate a numeric value (V) based on each criterion. Here, the alternatives set are signified by the decision matrix [$V_{ij}$], wherein $V_{ij}$ means the numeric value that shows how efficiently alternative $A_{x}$ could achieve criterion Cy.
  (a) The use of other source(s) of information, like available reports and statistics, or other quantitative or qualitative methods of gathering information for determination of V for additional criteria, with the use of the similar scale as Step (2).
  (b) Decision on the W for additional criteria based on the decision primacies by the decision-maker(s). This should be in comparison and balanced, with other $W_{x}$ from Step (1).
  (c) Normalizing the W matrix, as formulated in Equation (12). As clarified by Kien and Noraini [60], normalization is a method that translates all criteria into the corresponding dimension prior to relating with the weighted alternatives.
    (12) $$W_{x^{\prime }}=W_{x}/\sum W_{x}$$
(3) A weighted sum (WS) is derived after multiplying the weight for each criterion by a numeric value allocated to every alternative. The resulting value is summed, as shown in Equation (13).
  (13) $$\mathrm{WS}(A_{x})=(W_{x}\times V_{x})$$
(4) Equation (13) shows that for every Alternative ($A_{x}$), the WS could be derived after summing their respective resultant values.
  (14) $$\mathrm{WS}(A_{x})=\underset{y}{\sum }(\mathrm{WS}\left(A_{x})\right)$$
(5) Last, after comparing all the WS values, the alternatives which showed a maximum value, the best alternative for selection, and matched all the criteria could be derived from the most to the least preferred options.

### 2.2. Hybrid Decision-Making Method for HF Rearchitecting

Recently, researchers integrated two or more MCDM methods to overcome any limitations in one method (hybrid MCDM) [61,62,63]. Karami [64] and Zahidy [44] stated that decision-makers generally implement multiple DM techniques to make vital decisions and a gain better understanding of the problem. Zavadskas [62] mentioned that as the individual MCDM techniques yield differing rankings, the selection of an appropriate process was challenging. Hence, it is recommended to apply a hybrid approach, which uses multiple methods and then integrates the results for the final DM. 

To attain objective of this study, DEMATEL-WSM was employed as a hybrid MCDM method. Initially, DEMATEL was utilized based on the description in Section 2.1.1. In this research, DEMATEL can recognize the high-risk departments depending on the NIs risks caused by the interrelationship among the existing departments of a HF. Subsequently, the prioritizing of departments by DEMATEL was assessed using WSM, as described in Section 2.1.2. WSM can employ the listed managerial criteria of the selected department to determine the high-risk department(s) which should be excluded. 

### 2.3. Group Decision Making for HF Rearchitecting

Generally, the group decision-making (GDM) process includes many stakeholders who discuss the issue, list all alternatives after brainstorming, and reach a final consensus, which generates the final decisions [65]. The final outcome of the multiple-criteria GDM was seen to be more accurate than an individual decision-maker [66]. In this study, the researchers used an integrated technique of hybrid MCDMs and the GDM to solve the complicated DMs related to the HF rearchitecting. The stakeholders in this study included the HF managers, NI specialists, and the modification decision-makers. It was considered that the nominal group technique (NGT), from the GDM methods, is the adaptation of a brainstorming process where the DM group could offer their decisions individually, without any external interference, after noting them on paper or even electronically storing them [66,67]. During the last NGT stage, the electronic and paper contents were combined to generate the final ideas of the participants [67]. NGT is a method which could be implemented within a short time span and could be easily understood by all participants and applied practically to recognize the problems, idea development, and determination of the priorities of action [68]. Here, the researcher implemented the GDM, particularly the NGT.

A majority of the GDM and the consensus models consist of some scholars since, generally, the important decisions have to be made by the skilled or authorized people in the organizations, institutions, and administrations and professionals [68]. All scholars have their personal perspective, knowledge, drives, etc., are facing a common universal issue and are attempting to arrive at a common decision [69,70]. Here, every HF consisted of a different cluster, which included some scholars with a different area of expertise or knowledge in the field of decreasing the NI risks. To derive feasible results, it is important to determine how to weigh all the diverse clusters of the experts and then determine whose opinion is more valuable. Hence, one has to review the evaluation levels of the scholars in any group-decision analysis [71]. This study used a solution proposed earlier, wherein Sorooshian [66] recommended the nomination of different expert clusters based on their level of expertise, unbalanced expertise method. This weight of the opinions of every cluster was determined by the major decision-maker(s). In this study, the researchers applied the NGT technique, which included the experts with an unbalanced expertise level. This is known as the modified NGT, which is called NGT in the study.

### 2.4. The Process of Suggesting a New Algorithm for HF Rearchitecting

**Figure 1:**
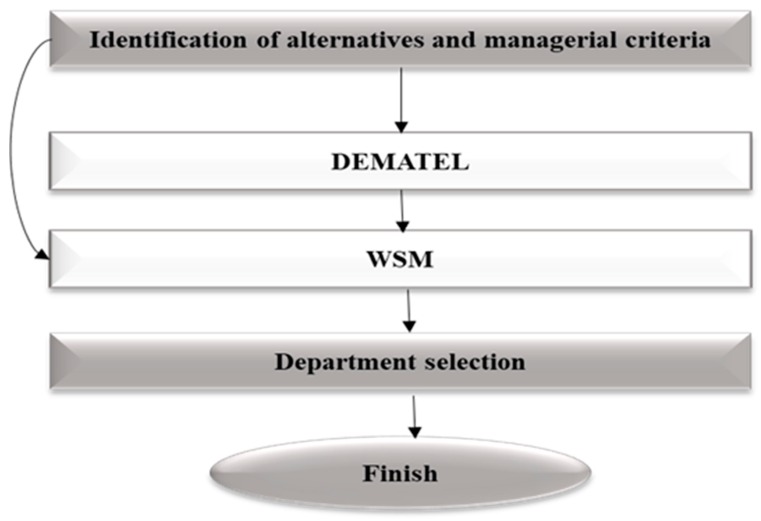
Flowchart of the rearchitecting of the healthcare facility (HF).

Through an integration of above hybrid techniques for HF rearchitecting, this research suggests a new DM algorithm in order to minimize the NI risks, as given below (illustrated in Figure 1).

Step 1: Alternative (existing departments) and managerial criteria identification using NGT.

Step 2: Computation by DEMATEL to identify and prioritize high-risk department(s) depending on NIs interrelationships between alternatives by use of NGT.

Step 3: Computation by WSM to identify department for excluding based on managerial criteria, from step 1, and NI risk, from Step 2.

Step 4: Selection of the potential department for rearchitecting.

### 2.5. Case Study

Creswell [72] defined a case study as the strategy of inquiry wherein a researcher can explore a research problem in depth, or any process, activity, event, or multiple individuals. Case studies are a valid and popular technique for testing the MCDM models [73,74]. Some researchers stated that conducting a case study was an important component of any research, which can resolve the following concerns [44]: Verify the result quality, assess all the analytical processes to determine their efficacy, and ensure all instruments are feasible and clear.

Along with the expert opinion, this study used a case study to test the feasibility and validity of the proposed HF department selection algorithm to minimize the risk of the NIs in the HFs. Hence, this algorithm could be used in the case study to derive an outcome, and the algorithm was presumed to be feasible. After conducting ample research for evaluating the potential HFs and validating the proposed algorithm, one HF in Iran agreed to cooperate and act as the case study in this report.

#### Characteristics of Case Study in this Research

The HF used as the case study in this report was established in 1949 and was the only accident and trauma HF in Kerman, a southeast Iranian province. Currently, it consists of 20 diagnostic and therapeutic departments, as listed below, and a total of 400 beds and 1400 medical staff. It also offers many student training programs, modern equipment-based therapeutic services, and other research and development-based programs. This HF was categorized as a public HF, which aimed to offer general education, along with a long-term and a tertiary-level care system.

ED1: Neurology1;
ED2: Neurology2;
ED3: General Surgery;
ED4: ICU1;
ED5: ICU2;
ED6: ICU3;
ED7: CCU;
ED8: Orthopedics (for men);
ED9: Orthopedics (for women);
ED10: Hematology/Oncology 1;
ED11: Hematology/Oncology 2;
ED12: Jaw and face surgery;
ED13: Urology;
ED14: Internal surgery;
ED15: Emergency;
ED16: Laboratory;
ED17: CT scanning;
ED18: Radiology;
ED19: Pathology;
ED20: Physiotherapy.

### 2.6. Data Collection

Here, to determine the transmission of NIs in the different HF departments, the researchers made some observations. The data was collected by conducting interviews with the management and the infection control experts to determine and analyze the management and the infection risk criteria before eliminating the high-risk HF departments. The interviews were conducted after applying the NGT method, and the data collected from every expert was ranked using an unbalanced expertise process. The final data was then used for testing the algorithm using various relative methods.

### 2.7. Nomination of Experts

To determine which experts to choose for collecting the data, based on the management guidance, the researchers interviewed the HF’s research authority as a study mentor. This mentor suggested the names of all potential experts who had a minimum of three years of work experience in the field of hospitals and also had ample knowledge about the management criteria and the NI risks. The mentor presented a list of 16 experts to the manager and hospital head for further approval. Among the list, 14 experts were approved, as shown in Table 1. Based on the description in Section 2.3, the approved experts were ranked based on their level of expertise in the case study using an unbalanced expertise method by the head and manager of the hospital with the maximal and minimal numbers, including 3 and 1, respectively, as shown in Table 1. After collecting all of the matrices for each expert, the rank for every expert was multiplied with the data of their matrix to enable further calculations.

### 2.8. Management Criteria Proposed by the Management Group for Rearchitecting the HF

To collect data, interviews were conducted using the NGT process. Here, the researchers determined the management criteria for eliminating all the high-risk HF departments by conducting open-ended interviews. Because of the unavailability and heavy workload of the selected experts, the data collection process was conducted for one month at the offices of every expert. The interviews were conducted for 30–60 min.

After completing the criteria collection process, the overlapping data were identified and represented as one criterion. To eliminate high-risk department(s) in the HF, 12 criteria were collected, respectively. The final list was analyzed by experts during the interview to score the criteria, and then all experts approved the list.

#### Collected Criteria for Eliminating High-Risk Department(s) from the HF

Here, 12 criteria were collected by conducting interviews with management experts, as listed below. For the analysis process, each managerial criterion for rearchitecting was named by MR1 to MR12.

MR1:  Lack of the corresponding expert (physician) in the department;
MR2: No correspondence of the department with hospital specialization and strategy;
MR3: Lack of demand and insignificance;
MR4: Lack of financial profitability;
MR5: High expenses of department maintenance;
MR6: Insufficient space for establishing and developing the department;
MR7: Dissatisfaction of patients from services;
MR8: Unavailability of facilities, equipment, and medication corresponding to treatment standards of every department (due to expenses, sanctions, etc.);
MR9: Lack of personnel (nursing, paramedic, etc.) to provide services;
MR10: Lack of correspondence with hospital construction standards (architecture) related to the department;
MR11: Ineffectiveness in enhancing training and research in order to improve hospital ranking based on MOH measures;
MR12: The inappropriateness of infrastructure conditions (wastewater and disinfection systems) as to department maintenance.

## 3. Results

### 3.1. Algorithm Implementation for HF ReArchitecting

By considering the algorithm process presented in Section 2.4, the subsections described in the data collection and analysis processes are explained. For rearchitecting the HFs, the proposed algorithm was implemented as described below. The various algorithm stages were described using Microsoft Excel 2016.

#### 3.1.1. Data Collection for DEMATEL and WSM

During the meeting, interviews were conducted with infection control experts, as presented in Table 1. The interviews were conducted in each expert’s office based on the NGT method. To run department elimination stages based on the algorithm described in Section 2.4, it was necessary to collect data for the DEMATEL method. Thus, each expert was asked closed-ended questions to weigh alternatives and assess the effect of existing departments on one another with regard to infection transmission based on infection risk criterion. Rates were assigned to each department depending on each department’s effectiveness in transmitting infection, beginning with 0 = no effect to 1 = low effect, 2 = moderate effect, 3 = high effect, and 4 = very high effect.

Also, regarding the data collection for the WSM, the interview with management experts, as shown in Table 1, was conducted to determine all the management criteria used to eliminate the high-risk departments based on the opinions presented by the experts during the interviews. Interview sessions were conducted in the offices of each expert using the NGT technique for 20–40 min. 

Each expert was asked many closed-ended questions to weigh all the management criteria from 0 to 100. This was based on the degree to which every criterion was vital for the selection of the potential departments that could be eliminated. The experts also had to explain the degree to which every criterion was important for eliminating the department, 0 to 100, wherein 100 was seen to be the most inappropriate, which led to a negative rating. The closer every criterion was to 100, the higher the probability was of eliminating that department.

#### 3.1.2. DEMATEL Matrix Analysis Obtained from Infection Control Experts in the HF for Rearchitecting based on Infection Risk Criterion

To analyze the matrix data obtained from infection experts based on the obtained rank for each expert presented in Table 1, the rank number of each was is multiplied into the data matrix of that expert, and the matrix average was obtained. The calculations for DEMATEL were conducted based on the descriptions in Section 2.1.1.

**Figure 2:**
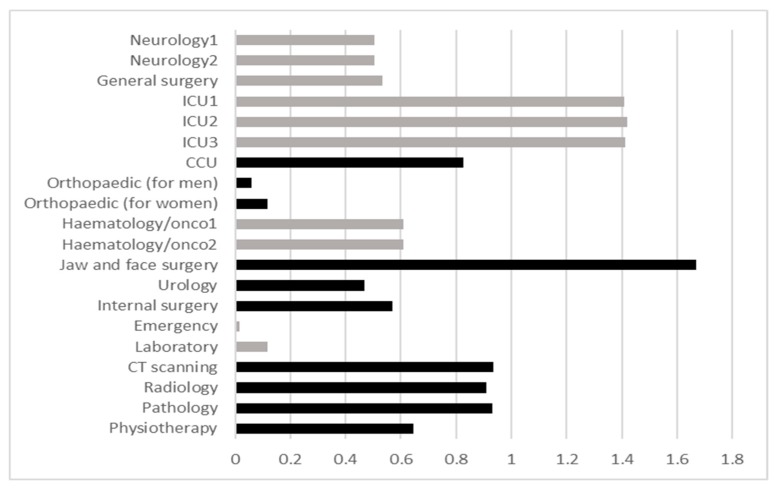
Measurement of infection risk for existing departments of the HF-based DEMATEL result.

After obtaining the total matrix at DEMATEL stage IV, the data total in every row (R) and data total in every column (C) were calculated. For each department, the R−C was calculated, and the analysis was done based on step V of DEMATEL. According to this step and based on Table 2, the cause and effect departments are shown in Figure 2. The cause group with a positive amount of R−C is indicated with gray bars and the effect group with a negative amount of R−C is indicated with black bars.

In the cause group (gray bars), ICU2 had impact on the other factors higher than the they influence on it, because it has higher positive amount of R-C in comparison to the other departments. In the effect group (black bars), jaw and face surgery because of its lower negative amount of R-C in comparison to the other departments is receiving the higher influence from the other factors of system. 

Therefore, ICU2 from the cause group and jaw and face surgery from the effect group were presented the high-risk existing departments of the HF in the field of cross-infection. Also, ICU1 and ICU3 were presented as high-risk departments because they had a few differences in the amounts of their R-C in comparison to ICU2. Introducing these departments may be contributive to the hospital and infection control authorities to reinforce infection control actions in these departments.

Based on the DEMATEL results matrix shown in Table 2, a chart (Figure 2) criterion of infection risk was considered for each department among 0–100. The numbers were placed in an extensive row at the end of the average matrix table of WSM from the management group to propose the elimination of a department or the increase of hygienic policies among them. By considering the algorithm process described in Section 2.4, it is necessary to run WSM method based on management criteria considered in eliminating a department obtained in interviews conducted with management experts.

#### 3.1.3. WSM Matrix Analysis for HF Rearchitecting Based on Management Criteria

Once the matrices were collected based on Section 3.1.1, after applying the unbalanced expertise method, the experts were assigned a ranking based on their expertise levels, as shown in Table 1. These assigned ranks were multiplied by the matrix of every expert and the average matrix was estimated and used for further calculations of the WSM method. 

Moreover, after determining the matrix averages, based on the DEMATEL results for every department, the researchers applied the different infection risk criteria along with the management criteria to eliminate the high-risk department in the HF, modified WSM. During the interview conducted with the hospital management, the row that corresponds to the infection risks in the matrix was not shown to the experts to reduce the bias noted in the results. The main objective of the study included the reduction of the NIs and also the determination of the significance of the NI risk zcriteria by the various infection control experts in an HF. Therefore, this criterion showed a weight value of 100 in the Column “a” of the WSM matrix. In the case of the other departments, weight values between 0 to 100 were assigned to the final WSM matrix in Table 3. The calculation processes were done based on the WSM stages in Section 2.1.2.

**Figure 3:**
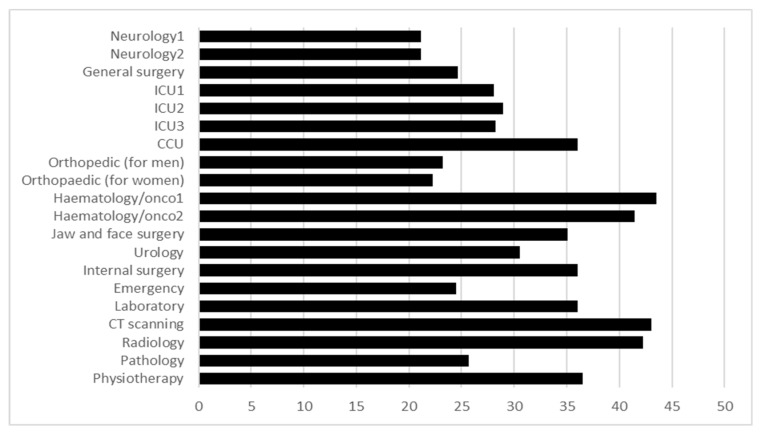
Result of WSM matrix from managerial experts for the rearchitecting of the HF.

After achieving total WSs for every alternative, all the results were compared to each other based on Figure 3, while higher ranks were given to the potential HF departments that must be proposed to eliminate or increase hygienic policies as per the opinions of the management experts. In this case, Hematology/Oncology 1, CT scanning, Radiology, and Hematology/Oncology 2 were suggested to the top managers of the HF to eliminate from the HF or increase their hygienic policies, respectively. The managers will make the final decision based on the results of the algorithm and their descriptions.

### 3.2. Final Analysis of Validity and Feasibility of Results obtained from Implementing the Algorithm in the HF as the Case of this Study

All results were presented and later approved by the manager and head of the HF, who stated that the study subject was very promising, and that all techniques were reasonable and comprehensive. After observing and analyzing all the results, the manager consented to this study, which was conducted to eliminate the high-risk HF departments for the purpose of reducing the NI risks, which is often missed by a majority of the therapeutic centers. The manager also stated that the methods were applicable, the results were very reasonable, the authors achieved the validity of the results. The manager stated that this algorithm could be applied to all the therapeutic centers, as they required infection control. Furthermore, this study could lead to the precise rearchitecting of the HFs, which could save a lot of time. Also, the fact that this algorithm was analyzed appropriately and that all results were acquired could establish the feasibility of the proposed algorithm. 

## 4. Discussion

Despite all the innovations and development seen in the healthcare sector and its management, a majority of the HFs still suffer from several health risks. NIs are major HF-related health risks which require more attention. Due to the infection related risks acquired from an HF environment, it is believed that the people are safer in their houses than in the HFs [75,76]. NIs are no longer a local issue, but a global problem [77]. The NIs increase the hospital stay of the patients, increase their resistance to the antimicrobials, and also increase the mortality rates of the patient [78]. The HF management and the public health authorities are aiming to find many cost-effective policies and strategies for preventing and controlling the NIs [78]. 

Khan [13] stated that though many attempts were made to control and prevent the NIs, the problem has not been completely eliminated. When proposing the solutions for all the issues, the HF layout and the department configuration was seen to be a contributive parameter. Significant evidence indicated that the HF plan layout was a major contributing factor which increases the NI transmission, and interventions related to the HF environment could mitigate the infection risk [7,79,80]. Currently, consent for controlling the infections is considered an important parameter which could improve the planning, construction, and the operations of the HFs [81]. The construction of the HFs is considered a hazardous task, as many pathogens can reside in the walls and the ceilings [26]. To improve the health and the safety of the society and decrease the risk of NIs, it is important to select and organize the departments and improve the layout of the HFs [82]. The layout configuration of the HFs was geared toward decreasing the costs and included many technologies [83]. The demolishment of the existent structures and construction of new improved structures was not seen to be a feasible solution to offer modern healthcare services and decrease the effects of the HF construction on the environment [29].

Hussain and Babalghith [82] stated that the major objective of improving the HFs was to maintain all positive aspects of the current HFs while improving the weaker aspects. One must consider the significance of the HF design in controlling the NIs and preventing their cross-infection among various departments. Hence, the HFs must be assessed to determine which of the departments need to be eliminated (rearchitecting), or which need to increase their hygienic policies based on their infection risk and other management criteria for preventing and reducing the outbreak of infection or intensifying the existing infections and their transmissions. Though the department selection and the layout configuration are vital for rearchitecting the HFs and controlling the transmission of the NIs, very few researchers have investigated the issue. Making final decisions related to the HF rearchitecting is seen to be a complicated task. 

Hence, there are very few systematic DM models established in this context. In this study, the researchers proposed a new algorithm, which could contribute to modelling and rearchitecting the HFs for controlling the NI transmission. 

The hybridization and the DM algorithm used in this study were seen to be a pioneer. This study introduced a novel DEMATEL-WSM technique (based on a modified NGT). According to Tzeng and Shen [84], the novel hybrid MCDM, along with the rankings or selection, could be used for improving the performance limitations of the current MCDMs and all the subsequent aspects.

## 5. Conclusions

This study is a quantitative research and based on Stefanini, A. [85] categorization, the research focused particularly on a strategic level in healthcare service planning, which had a large impact on performance measures, such as service quality, patient safety, and etcetera. The main objective of the present research, which attempted to decrease NIs risks that affected patient safety and service quality, may be the other reason that this study worked on a strategic level. Also, the author mentioned that most of the approaches in quantitative researches at a strategic level in healthcare service planning are very time-consuming, like simulation studies and analytical approaches, and data collection is expensive, like simulation studies. However, the proposed algorithm of present study requires less resources, such as time, budget, and skill, in comparison with the other methodologies. Also, the mathematical process of algorithm is not confusing, but valid. 

Although this study was conducted in a strategic level of HF management, the operational variables that carried weight in the onset of infections, such as the criteria for air filtration, cleaning surfaces, and washing hands, should not be neglected. The rate of infection for each facility varied based on the practice of these factors. To consider the prevention and control operational practices, the panel of experts for data collection should be selected by top managers of HF and based on their knowledge of the current infection statistics and operational practices of the HF.

## Figures and Tables

**Table 1:** List of experts and their ranking based on an unbalanced expertise method.

Categorization of Experts	Position	Duration of Professional Experience	Specialty	Ranking No.
Experts related to assessing infection risks	The HF infection expert and member of infection control	7 years	Ph.D. in Infectious disease specialist	3
	The HF infection control coordinator	8 years	M.Sc. of Nursing	3
	The HF infection expert and member of infection control	5 years	Ph.D. in Infectious disease specialist	3
	The HF infection expert and member of infection control	3 years	Ph.D. in Infectious disease specialist	3
	The HF nursing head	11 years	M.Sc. of Nursing	2
	The HF quality improvement committee coordinator	7 years	M.Sc. of management	2
	Environment health coordinator	6 years	B.Sc. of environmental health	1
Experts related to collecting management criteria	Head of the HF	12 years	Ph.D. in Anesthesia and Fellowship Specialist of ICU	3
	Manager of the HF	15 years	Ph.D. in Internal disease specialist	3
	The HF development committee coordinator	3 years	B.Sc in Engineering	2
	The HF quality improvement committee coordinator	7 years	M.Sc. of management	2
	The HF crisis and hazard committee authority	6 years	M.Sc. of management	2
	Research coordinator	4 years	M.Sc. of Nursing	1
	Training coordinator	4 years	B.Sc. of Nursing	1

Note: HF—healthcare facility.

**Table 2:** Matrix of Decision-Making Trial and Evaluation Laboratory (DEMATEL) to evaluate interrelationships among existing departments based on infection risk for the rearchitecting of the Bahonar hospital.

Departments	ED20	ED19	ED18	ED17	ED16	ED15	ED14	ED13	ED12	ED11	ED10	ED9	ED8	ED7	ED6	ED5	ED4	ED3	ED2	ED1	R	R-C
**ED20**	0.0133	0.0247	0.0144	0.0138	0.01	0.02	0.03	0.03	0.02	0.03	0.03	0.02	0.0278	0.03	0.04	0.04	0.04	0.03	0.03	0.0306	**0.5965**	**−0.647**
**ED19**	0.0100	0.0212	0.0109	0.0103	0.02	0.01	0.03	0.02	0.01	0.03	0.03	0.01	0.0157	0.02	0.02	0.02	0.02	0.01	0.02	0.0279	**0.4613**	**−0.930**
**ED18**	0.0094	0.0106	0.0139	0.0098	0.01	0.03	0.03	0.02	0.02	0.02	0.02	0.03	0.0217	0.02	0.02	0.02	0.02	0.02	0.02	0.0240	**0.4537**	**−0.910**
**ED17**	0.0077	0.0085	0.0085	0.0114	0.00	0.02	0.02	0.02	0.02	0.02	0.02	0.01	0.0190	0.02	0.02	0.02	0.02	0.02	0.02	0.0207	**0.3622**	**−0.934**
**ED16**	0.0440	0.0476	0.0471	0.0454	0.06	0.08	0.08	0.08	0.08	0.09	0.09	0.07	0.0775	0.09	0.09	0.09	0.10	0.08	0.08	0.0850	**1.6005**	**0.1158**
**ED15**	0.0637	0.0791	0.0817	0.0690	0.08	0.13	0.12	0.12	0.11	0.13	0.13	0.10	0.1017	0.12	0.13	0.13	0.13	0.11	0.11	0.1119	**2.2539**	**0.0156**
**ED14**	0.0505	0.0649	0.0643	0.0521	0.06	0.09	0.11	0.10	0.10	0.12	0.12	0.08	0.0841	0.10	0.11	0.11	0.11	0.09	0.09	0.0994	**1.8993**	**−0.569**
**ED13**	0.0509	0.0655	0.0649	0.0627	0.06	0.10	0.11	0.12	0.10	0.13	0.13	0.08	0.0849	0.11	0.11	0.11	0.11	0.09	0.09	0.0936	**1.9511**	**−0.469**
**ED12**	0.0114	0.0126	0.0125	0.0119	0.01	0.02	0.03	0.03	0.03	0.03	0.03	0.02	0.0282	0.03	0.03	0.03	0.03	0.03	0.03	0.0308	**0.5358**	**−1.669**
**ED11**	0.1050	0.1126	0.1114	0.1078	0.11	0.16	0.18	0.18	0.17	0.19	0.19	0.15	0.1539	0.17	0.17	0.17	0.17	0.16	0.16	0.1685	**3.1807**	**0.6089**
**ED10**	0.1050	0.1126	0.1114	0.1078	0.11	0.16	0.18	0.18	0.17	0.19	0.19	0.15	0.1539	0.17	0.17	0.17	0.17	0.16	0.16	0.1685	**3.1807**	**0.6089**
**ED9**	0.0517	0.0562	0.0557	0.0534	0.06	0.10	0.11	0.11	0.09	0.12	0.12	0.09	0.0828	0.11	0.11	0.11	0.11	0.11	0.11	0.1118	**1.9383**	**−0.116**
**ED8**	0.0517	0.0562	0.0557	0.0534	0.06	0.10	0.11	0.11	0.08	0.12	0.12	0.08	0.0895	0.11	0.11	0.11	0.11	0.11	0.11	0.1118	**1.9383**	**−0.058**
**ED7**	0.0439	0.0476	0.0471	0.0453	0.04	0.08	0.09	0.09	0.06	0.09	0.09	0.07	0.0635	0.09	0.09	0.09	0.09	0.09	0.09	0.0909	**1.5729**	**−0.824**
**ED6**	0.1204	0.1294	0.1280	0.1237	0.13	0.20	0.22	0.22	0.20	0.23	0.21	0.19	0.1917	0.22	0.22	0.22	0.22	0.21	0.21	0.2132	**3.8839**	**1.4116**
**ED5**	0.1206	0.1297	0.1283	0.1240	0.13	0.20	0.22	0.22	0.20	0.23	0.23	0.19	0.1921	0.22	0.22	0.22	0.22	0.21	0.21	0.2136	**3.8916**	**1.4193**
**ED4**	0.1206	0.1297	0.1283	0.1240	0.13	0.20	0.22	0.22	0.20	0.23	0.23	0.19	0.1921	0.22	0.22	0.22	0.22	0.21	0.21	0.2136	**3.8917**	**1.4092**
**ED3**	0.0875	0.0939	0.0929	0.0898	0.09	0.14	0.15	0.15	0.14	0.16	0.16	0.14	0.1385	0.15	0.15	0.15	0.16	0.15	0.15	0.1515	**2.7805**	**0.5329**
**ED2**	0.0875	0.0940	0.0930	0.0899	0.09	0.14	0.15	0.15	0.14	0.16	0.16	0.14	0.1386	0.15	0.16	0.16	0.16	0.15	0.15	0.1550	**2.7858**	**0.5041**
**ED1**	0.0875	0.0940	0.0930	0.0899	0.09	0.14	0.15	0.15	0.14	0.16	0.16	0.14	0.1386	0.15	0.16	0.16	0.16	0.15	0.15	0.1583	**2.7858**	**0.5041**
**C**	**1.2435**	**1.3916**	**1.3638**	**1.2963**	**1.48**	**2.23**	**2.46**	**2.42**	**2.20**	**2.57**	**2.57**	**2.05**	**1.9971**	**2.39**	**2.47**	**2.47**	**2.48**	**2.24**	**2.28**	**2.2816**		

Note: ED—existing departments; C—column; R—row.

**Table 3:** Result of weighted sum method (WSM) matrix for removing departments.

Existing Departments Managerial Criterion	Weigh for Each Criterion “a”	ED20	ED19	ED18	ED17	ED16	ED15	ED14	ED13	ED12	ED11	ED10	ED9	ED8	ED7	ED6	ED5	ED4	ED3	ED2	ED1
**MR1**	**0.07211**	0.86	0.288	2.163	1.874	2.307	2.019	2.163	0	0.288	0.288	0.288	0	0	0.865	0	0	0	0	0	0
**MR2**	**0.11057**	0	0	0	0	0	0	1.769	2.211	0.884	7.961	7.961	0	0	1.326	0	0	0	0	0	0
**MR3**	**0.10817**	3.02	0.216	0	0	1.307	0	3.894	4.110	4.110	0.432	0.432	0	0	0.432	0	0	0	0	0	0
**MR4**	**0.10336**	4.13	4.548	5.374	7.235	5.788	2.894	7.028	1.033	1.860	6.408	6.408	0.413	0.413	4.961	1.653	1.653	1.653	0.826	0.826	0.826
**MR5**	**0.05528**	2.98	2.764	5.086	5.307	3.759	2.543	1.105	2.874	2.985	2.874	2.874	2.874	2.874	3.538	4.644	4.644	4.644	2.764	2.874	2.874
**MR6**	**0.06971**	3.34	1.812	3.903	3.346	3.625	1.673	2.927	4.043	3.206	2.927	2.788	2.509	4.461	4.043	2.230	2.230	2.230	2.788	2.788	2.788
**MR7**	**0.06971**	3.62	1.115	3.625	3.625	1.673	3.067	1.394	1.673	1.394	3.067	3.067	1.951	2.509	0.557	0.557	0.557	0.557	3.067	1.115	1.115
**MR8**	**0.04086**	1.71	0.735	2.043	1.552	2.206	1.144	1.552	1.961	1.389	3.269	3.269	1.634	1.798	2.124	1.798	1.798	1.798	1.634	1.798	1.798
**MR9**	**0.06490**	3.24	2.076	4.413	3.634	3.375	3.894	2.596	2.076	1.817	2.206	2.206	2.855	3.115	1.298	2.336	2.336	2.336	3.245	2.855	2.855
**MR10**	**0.40865**	3.26	1.798	2.778	2.778	2.533	2.615	2.124	2.288	2.043	2.370	3.514	2.615	1.879	1.225	2.288	2.124	2.124	1.471	1.389	1.389
**MR11**	**0.09855**	2.16	0.591	3.350	3.350	3.350	1.576	1.971	1.576	1.182	2.365	2.365	1.576	1.576	6.701	0.788	0.788	0.788	1.971	1.182	1.182
**MR12**	**0.04567**	1.55	1.552	1.826	2.192	2.100	1.826	2.100	1.918	1.918	1.278	2.374	2.192	2.192	1.278	1.826	2.009	1.735	1.461	1.461	1.461
**Infection Risk**	**0.12019**	6.61	8.173	7.692	8.173	3.605	1.201	5.408	4.807	12.01	6.009	6.009	3.605	2.403	7.692	10.09	10.81	10.21	5.408	4.807	4.807
**Ws**	-	**36.5**	**25.67**	**42.25**	**43.07**	**36.05**	**24.45**	**36.03**	**30.57**	**35.10**	**41.46**	**43.56**	**22.23**	**23.22**	**36.04**	**28.22**	**28.96**	**28.08**	**24.63**	**21.10**	**21.10**

Note: ED—existing departments; MR—managerial criterion; Ws—weighted sum.
